# Effect of smoking on the development of chronic obstructive pulmonary disease in young individuals: a nationwide cohort study

**DOI:** 10.3389/fmed.2023.1190885

**Published:** 2023-08-01

**Authors:** Chiwook Chung, Kyu Na Lee, Kyungdo Han, Dong Wook Shin, Sei Won Lee

**Affiliations:** ^1^Department of Pulmonary and Critical Care Medicine, Asan Medical Center, University of Ulsan College of Medicine, Seoul, Republic of Korea; ^2^Department of Pulmonary, Allergy, and Critical Care Medicine, Gangneung Asan Hospital, University of Ulsan College of Medicine, Gangneung, Republic of Korea; ^3^Department of Statistics and Actuarial Science, Soongsil University, Seoul, Republic of Korea; ^4^Supportive Care Center, Samsung Comprehensive Cancer Center, Department of Family Medicine, Samsung Medical Center, Sungkyunkwan University School of Medicine, Seoul, Republic of Korea

**Keywords:** cigarette smoking, smoker, young age, female, COPD

## Abstract

**Background:**

Cigarette smoking is an important risk factor for developing chronic obstructive pulmonary disease (COPD). However, the effect of smoking on the development of COPD in young individuals remains unclear. We aimed to evaluate the effect of smoking on COPD development in young individuals.

**Methods:**

Using the Korean National Health Information Database, we screened individuals aged 20–39 years who participated in the national health check-up between 2009 and 2012. We defined physician-diagnosed COPD based on health insurance claims and searched the database until December 2019. We identified 6,307,576 eligible individuals, and 13,789 had newly developed COPD. We used multivariate Cox proportional hazards models to estimate the adjusted hazard ratio (aHR) of risk factors for COPD.

**Results:**

The incidence rate for developing COPD was 0.26/1000 person-year. The risk of developing COPD was significantly higher in current smokers [aHR 1.46, 95% confidence interval (CI) 1.39–1.53] and former smokers (aHR 1.21, 95% CI 1.14–1.29) than in non-smokers. Furthermore, the risk increased with increasing smoking amounts (≥20 pack-years, aHR 2.24; 10–20 pack-years, aHR 1.55; <10 pack-years, aHR 1.27). Female participants had a higher relative risk of developing COPD due to smoking, compared with their male counterparts.

**Conclusion:**

Cigarette smoking increased the risk of developing COPD in young individuals. Current and heavy smokers had higher risks of developing COPD than non-smokers. Female smokers were more likely to develop COPD than male smokers.

## Introduction

Chronic obstructive pulmonary disease (COPD) is a chronic respiratory disease with persistent airflow limitation usually caused by prolonged exposure to noxious gases or particles, particularly cigarette smoke (1, 2). Important risk factors for developing COPD include male, advanced age, cigarette smoking, low body mass index (BMI), biomass fuel or occupational smoke exposure, childhood respiratory illness, asthma, tuberculosis, low socioeconomic status, and low education level (1, 2).

COPD is associated with lung aging, and its prevalence increases with age; thus, it is considered as a disease of elderly people (2, 3). However, some individuals under 50 years of age have been diagnosed with COPD (4–6), and this category of patients has been named “Young COPD” (4), “Early-onset COPD” (5), or “Early COPD” (6). In 2019, it was estimated that 49.3 million people aged 30–39 years had COPD worldwide, corresponding to a prevalence of approximately 4% (3.8% in 30–34 years, and 4.9% in 35–39 years) (2).

Cigarette smoking has been proposed as a main cause of COPD also in young population (7, 8). The prevalence of cigarette smoking in American adolescents and early adulthood has recently increased, owing to electronic cigarette (e-cigarette) usage (9, 10). Thus, individuals aged 20–30 years smoked substantial amounts of cigarette (>10 pack-years) (6), which may have caused COPD. However, most studies on COPD enrolled patients aged 40 years or older. Previous studies on young individuals with COPD addressed limited cohort populations and lacked dose-relationships and sexual differences of smoking for COPD development (7, 8, 11–13). Thus, the effect of smoking on the development of COPD in young individuals remains unclear. Therefore, we evaluated whether smoking is a considerable risk factor for COPD development in young individuals (<40 years) by identifying the effects of cigarette smoking on COPD development using the Korean National Health Information Database (NHID).

## Methods

### Data source and study design

The NHID is a public database that provides health check-up, health care use, socio-demographic data, and mortality for the entire population of the Republic of Korea (14). The database was formed by the National Health Insurance Service (NHIS), which is a national healthcare insurance service managed by the government (14). The NHID contains personal data, demographics, medical treatment and claims information, long-term care insurance, and national health check-up program databases (14, 15). The NHIS has provided national health check-up services for the early detection and prevention of diseases since 1995 (16). Until 2018, all adults aged ≥40 years and adult employees regardless of age were eligible to a biennial health check-up (annually for manual workers), including chest radiograph, laboratory tests, and questionnaire about lifestyle habits and medical history (15, 17).

This was a retrospective nationwide cohort study that included individuals aged 20–39 years who participated in the national health check-up service between 2009 and 2012. Thereafter, we searched their medical claims through the NHID until December 2019. This study protocol was approved by the Institutional Review Board of the Asan Medical Center, Seoul, Republic of Korea (approval number 2022-1593). The requirement for informed consent was waived, as it was a retrospective study and the data used were anonymized. This study complied with the guidelines stipulated in the Declaration of Helsinki, and all methods were performed in accordance with the relevant guidelines.

### Study population

We defined newly diagnosed COPD as follows: (1) International Classification of Diseases 10th Revision (ICD-10) codes for COPD (J44.x) or emphysema (J43.x), except for J43.0 (unilateral pulmonary emphysema, Macleod’s syndrome); (2) medical insurance claims for the aforementioned codes more than 3 times/year for at least 2 years (more than 3 times/year for each year) (18, 19).

**Figure 1:**
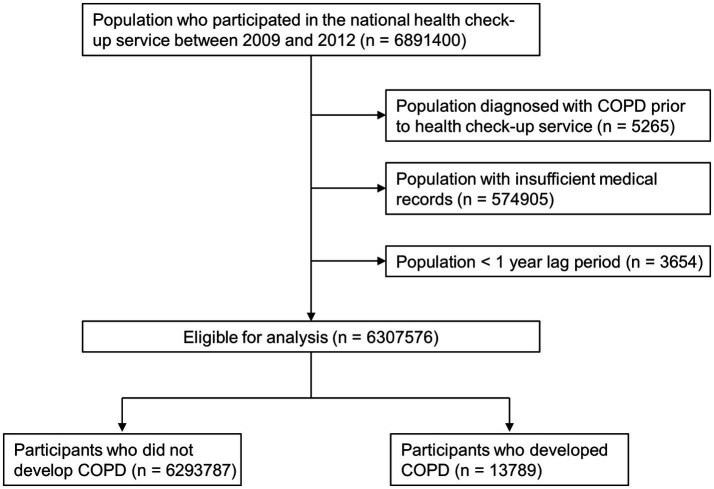
Flow chart of the study population. COPD, chronic obstructive pulmonary disease.

Our search of the NHID revealed 6,891,400 individuals aged 20–39 who participated in the national health check-up service provided by the NHIS between 2009 and 2012. We excluded 5,265 individuals with any medical claim with ICD-10 codes for COPD prior to their health check-up, 574,905 individuals with insufficient medical records, and 3,654 individuals diagnosed with COPD within 1 year after index date (<1 year lag period). As a result, 6,307,576 participants were included in this study. Furthermore, we assessed their medical claim data using the NHID until December 2019 and identified 13,789 participants who newly developed COPD (Figure 1).

### Covariates

Participants’ lifestyle information was collected from self-reported questionnaires during health check-ups, which included the following data: daily smoking amount and duration (1 pack-year: 20 cigarettes smoked daily for 1 year), smoking status (non-, former, or current smokers), physical activity, and alcohol consumption. We defined a former smoker as a person who had smoked at least 100 cigarettes or cigars during lifetime but did not smoke at the time of health check-up. BMI was calculated by dividing the body weight by height squared (kg/m^2^). Income level was categorized into quartiles (Q1 = lowest income and Q4 = highest income) based on the payer’s annual national health insurance premium. Regular exercise was defined as >30 min of moderate physical activity at least 5 times/week or >20 min of strenuous physical activity at least 3 times/week (20). Alcohol consumption was classified as none, mild (<30 g/day), or heavy (≥30 g/day) (21).

Comorbidities were identified using NHIS claims data and laboratory data from the national health check-up service. Diabetes mellitus was indicated by either the record of ICD-10 codes E11–14 (with the prescription of hypoglycemic medication) or a fasting serum glucose level of ≥126 mg/dL in the health check-up data. Hypertension was indicated by either the record of ICD-10 codes I10–13 and I15 (with the prescription of antihypertensive medication) or high blood pressure (a systolic blood pressure of ≥140 mmHg or a diastolic blood pressure of ≥90 mmHg) records in the health check-up data. Dyslipidemia was indicated by either a record of the ICD-10 code E78 (with the prescription of lipid-lowering medication) or a record of total serum cholesterol levels of ≥240 mg/dL in the health check-up data.

History of respiratory illness is an important risk factor for the development of COPD (2, 22, 23). We defined a history of pneumonia as follows: (1) ICD-10 codes for pneumonia, namely, J10.0, J11.0, J12–J18, or A481; (2) hospitalization for the aforementioned codes; and (3) within 5 years prior to COPD diagnosis. We defined a history of asthma as follows: (1) ICD-10 codes for asthma, namely, J45; (2) medical insurance claims for the aforementioned codes more than 3 times/year; and (3) within 5 years prior to COPD diagnosis.

### Statistical analysis

Continuous variables are presented as mean ± standard deviation, and categorical variables are expressed as numbers (percentage). Student’s *t*-test and *χ*^2^ test were used to compare continuous and categorical variables, respectively. COPD incidence rates were calculated as the ratio between the number of newly-developed COPD cases and the number of person-years at risk of COPD (per 1,000). Multivariate Cox proportional hazards model was used to identify risk factors for the development of COPD. Model 1 was not adjusted. In model 2 (the main analysis model), the covariates included were age, sex, BMI, alcohol consumption, regular exercise, income, pneumonia, and asthma. Model 3 contained the covariates in Model 2 as well as diabetes mellitus, hypertension, and dyslipidemia. In Models 2 and 3, stratification analyses by sex were performed to determine the associations between smoking and COPD development. Subgroup analyses for interaction were performed for Model 2. All value of *p*s were two-tailed, with statistical significance set at *p* < 0.05. All statistical analyses were performed using SAS V.9.4 (SAS institute, Cary, NC, United States).

## Results

### Baseline characteristics of the study population

The mean age of all participants was 30.84 years, with men accounting for 59.21%. The participants in the COPD group were significantly older than those in the non-COPD group (32.6 ± 4.72 vs. 30.84 ± 5.00, *p* < 0.0001), and men were predominant (63.13%). In the COPD group, 5,626 (40.8%) were current smokers and 1,536 (11.14%) were former smokers. Furthermore, approximately 70% of men and 10% of women had a smoking history (Table 1; Supplementary Table S1).

**Table 1:** Baseline characteristics of study population.

*N*	Total	Non-COPD	COPD	Value of *p*
	6,307,576	6,293,787	13,789	
Age, years	30.84 ± 5	30.84 ± 5	32.6 ± 4.72	<0.0001
Age groups				<0.0001
<30 years	2,669,087 (42.32)	2,665,337 (42.35)	3,750 (27.2)	
≥30 years	3,638,489 (57.68)	3,628,450 (57.65)	10,039 (72.8)	
Sex				<0.0001
Male	3,734,883 (59.21)	3,726,178 (59.2)	8,705 (63.13)	
Female	2,572,693 (40.79)	2,567,609 (40.8)	5,084 (36.87)	
BMI, kg/m^2^	22.99 ± 3.61	22.99 ± 3.61	23.17 ± 3.88	<0.0001
Income, lowest Q1*	1,367,132 (21.67)	1,364,211 (21.68)	2,921 (21.18)	0.1613
Smoking status				<0.0001
Non	3,485,089 (55.25)	3,478,462 (55.27)	6,627 (48.06)	
Former	633,122 (10.04)	631,586 (10.04)	1,536 (11.14)	
Current	2,189,365 (34.71)	2,183,739 (34.7)	5,626 (40.8)	
Alcohol consumption				<0.0001
Non	2,388,341 (37.86)	2,382,782 (37.86)	5,559 (40.31)	
Mild	3,364,896 (53.35)	3,357,914 (53.35)	6,982 (50.63)	
Heavy	554,339 (8.79)	553,091 (8.79)	1,248 (9.05)	
Regular exercise	807,860 (12.81)	806,090 (12.81)	1770 (12.84)	0.92
Diabetes mellitus	122,207 (1.94)	121,789 (1.94)	418 (3.03)	<0.0001
Hypertension	2,239,408 (35.5)	2,234,091 (35.5)	5,317 (38.56)	<0.0001
Dyslipidemia	433,783 (6.88)	432,558 (6.87)	1,225 (8.88)	<0.0001
CKD	170,405 (2.7)	170,032 (2.7)	373 (2.71)	0.98
Asthma	182,672 (2.9)	180,518 (2.87)	2,154 (15.62)	<0.0001
Pneumonia	26,548 (0.42)	26,293 (0.42)	255 (1.85)	<0.0001

Data are presented as the mean ± standard deviation or number (%), unless otherwise indicated. *First quartile of medical insurance premiums and medical aid beneficiaries. COPD, chronic obstructive pulmonary disease; BMI, body mass index; CKD, chronic kidney disease. CKD was defined by either a record of ICD-10 codes N18–19 or an estimated glomerular filtration rate of <60 mL/min/1.73 m^2^ using the Modification of Diet in Renal Disease equation in the health check-up data.

### Risk of COPD development based on smoking status

The incidence of developing COPD was 0.2288/1000 person-year (PY) in non-smokers, 0.2862/1000 PY in former smokers, and 0.3063/1000 PY in current smokers. The risk of developing COPD was higher in former smokers [adjusted hazard ratio (aHR) 1.208, 95% confidence interval (CI) 1.135–1.285] and the highest in current smokers (aHR 1.460, 95% CI 1.394–1.529) in Model 2. As for sexual differences, non-smokers had similar incidence rates regardless of sex (0.2282/1000 PY in men vs. 0.2291/1000 PY in women); however, female smokers had higher incidence and risks of developing COPD (Table 2; Supplementary Table S2). In the subgroup analysis, age, BMI, alcohol consumption, and asthma history showed significant associations between smoking status and COPD development (Supplementary Table S3).

**Table 2:** Impact of smoking status on COPD development.

Sex	Smoking status	*N*	Event	Duration	IR, per 1,000 PY	HR (95% CI)		
						Model 1	Model 2	Model 3
Total	Non	3,485,089	6,627	28963394.53	0.2288	1 (Ref.)	1 (Ref.)	1 (Ref.)	Former	633,122	1,536	5367167.29	0.2862	1.247 (1.180–1.318)	1.208 (1.135–1.285)	1.206 (1.134–1.283)	Current	2,189,365	5,626	18369092.08	0.3063	1.336 (1.290–1.385)	1.460 (1.394–1.529)	1.455 (1.389–1.524)
Male*	Non	1,148,534	2,192	9605130.78	0.2282	1 (Ref.)	1 (Ref.)	1 (Ref.)	Former	547,055	1,320	4670421.89	0.2826	1.235 (1.153–1.322)	1.116 (1.041–1.197)	1.115 (1.040–1.195)	Current	2,039,294	5,193	17160746.25	0.3026	1.324 (1.260–1.392)	1.395 (1.324–1.469)	1.390 (1.320–1.464)
Female*	Non	2,336,555	4,435	19358263.75	0.2291	1 (Ref.)	1 (Ref.)	1 (Ref.)	Former	86,067	216	696745.4	0.3100	1.358 (1.185–1.557)	1.389 (1.210–1.595)	1.388 (1.210–1.594)	Current	150,071	433	1208345.83	0.3583	1.573 (1.425–1.736)	1.658 (1.497–1.837)	1.654 (1.493–1.833)

Model 1, non-adjusted; Model 2, adjusted for age, sex, BMI, alcohol consumption, regular exercise, income, pneumonia, asthma; Model 3, adjusted for age, sex, BMI, alcohol consumption, regular exercise, income, pneumonia, asthma, diabetes mellitus, hypertension, dyslipidemia. *Sex was excluded from multivariate models. COPD, chronic obstructive pulmonary disease; IR, incidence rate; PY, person-year; HR, hazard ratio; CI, confidence interval; Ref., reference.

### Risk of COPD development based on smoking amount

The incidence of COPD increased with smoking amount, resulting in the highest incidence rate (0.5971/1000 PY) in individuals with ≥20 pack-years of smoking history. They also had the highest risk (aHR 2.236, 95% CI 2.062–2.425) for developing COPD compared to non-smokers in Model 2. Female smokers had higher incidence and risks of developing COPD than male smokers (Table 3; Supplementary Table S4).

**Table 3:** Impact of smoking amount on COPD development.

Sex	Smoking amount, Pack-year	*N*	Event	Duration	IR, per 1,000 PY	HR (95% CI)		
						Model 1	Model 2	Model 3
Total	Non	3,485,089	6,627	28963394.53	0.2288	1 (Ref.)	1 (Ref.)	1 (Ref.)	<10	1,849,834	3,889	15455833.55	0.2516	1.099 (1.056–1.143)	1.266 (1.206–1.328)	1.264 (1.205–1.327)	10 ≤ … < 20	803,870	2,416	6845253.4	0.3530	1.537 (1.467–1.610)	1.548 (1.461–1.639)	1.539 (1.453–1.631)	≥20	168,783	857	1435172.42	0.5971	2.599 (2.421–2.791)	2.236 (2.062–2.425)	2.209 (2.037–2.396)
Male*	Non	1,148,534	2,192	9605130.78	0.2282	1 (Ref.)	1 (Ref.)	1 (Ref.)	<10	1,623,329	3,294	13626880.68	0.2417	1.059 (1.003–1.117)	1.177 (1.113–1.244)	1.175 (1.112–1.242)	10 ≤ … < 20	795,199	2,370	6776623.49	0.3497	1.528 (1.441–1.619)	1.456 (1.370–1.547)	1.450 (1.365–1.541)	≥20	167,821	849	1427663.97	0.5947	2.597 (2.399–2.811)	2.067 (1.899–2.249)	2.049 (1.883–2.231)
Female*	Non	2,336,555	4,435	19358263.75	0.2291	1 (Ref.)	1 (Ref.)	1 (Ref.)	<10	226,505	595	1828952.87	0.3253	1.426 (1.309–1.553)	1.508 (1.380–1.648)	1.506 (1.378–1.646)	10 ≤ … < 20	8,671	46	68629.92	0.6703	2.946 (2.204–3.940)	2.346 (1.749–3.146)	2.320 (1.730–3.112)	≥20	962	8	7508.44	1.0655	4.696 (2.347–9.394)	3.265 (1.627–6.552)	3.193 (1.591–6.408)

Model 1, non-adjusted; Model 2, adjusted for age, sex, BMI, alcohol consumption, regular exercise, income, pneumonia, asthma; Model 3, adjusted for age, sex, BMI, alcohol consumption, regular exercise, income, pneumonia, asthma, diabetes mellitus, hypertension, dyslipidemia. *Sex was excluded from multivariate models. COPD, chronic obstructive pulmonary disease; IR, incidence rate; PY, person-year; HR, hazard ratio; CI, confidence interval; Ref., reference.

### Risk of COPD development based on smoking status and amount

Regardless of smoking status, the incidence of COPD increased with smoking amount, resulting in the highest incidence rate (0.5086/1000 PY) in current smokers with ≥15 pack-years of smoking history. They also had the highest risk (aHR 2.015, 95% CI 1.888–2.150) of developing COPD compared to non-smokers in Model 2. Moreover, female smokers had relatively higher risks of developing COPD than male smokers given the same smoking status and amount (Table 4; Supplementary Table S5).

**Table 4:** Impact of smoking status and amount on COPD development.

Sex	Smoking status and amount, Pack-year	*N*	Event	Duration	IR, per 1,000 PY	HR (95% CI)		
						Model 1	Model 2	Model 3
Total	Non	3,485,089	6,627	28963394.53	0.2288	1 (Ref.)	1 (Ref.)	1 (Ref.)	Former and <15	572,247	1,321	4843386.08	0.2727	1.189 (1.121–1.261)	1.201 (1.125–1.281)	1.200 (1.124–1.280)	Former and ≥15	60,875	215	523781.21	0.4105	1.785 (1.558–2.044)	1.493 (1.297–1.718)	1.478 (1.284–1.702)	Current and <15	1,805,482	3,966	15104966.38	0.2626	1.146 (1.102–1.192)	1.339 (1.275–1.406)	1.336 (1.272–1.403)	Current and ≥15	383,883	1,660	3264125.7	0.5086	2.215 (2.099–2.337)	2.015 (1.888–2.150)	1.997 (1.872–2.132)
Male*	Non	1,148,534	2,192	9605130.78	0.2282	1 (Ref.)	1 (Ref.)	1 (Ref.)	Former and <15	486,675	1,110	4150558.81	0.2674	1.168 (1.087–1.256)	1.104 (1.026–1.189)	1.104 (1.026–1.188)	Former and ≥15	60,380	210	519863.08	0.4040	1.762 (1.529–2.030)	1.369 (1.185–1.581)	1.360 (1.177–1.570)	Current and <15	1,657,807	3,546	13915396.11	0.2548	1.116 (1.058–1.177)	1.262 (1.194–1.333)	1.259 (1.192–1.330)	Current and ≥15	381,487	1,647	3245350.14	0.5075	2.217 (2.079–2.363)	1.876 (1.752–2.010)	1.865 (1.741–1.998)
Female*	Non	2,336,555	4,435	19358263.75	0.2291	1 (Ref.)	1 (Ref.)	1 (Ref.)	Former and <15	85,572	211	692827.28	0.3046	1.334 (1.162–1.532)	1.369 (1.191–1.573)	1.368 (1.190–1.572)	Former and ≥15	495	5	3918.12	1.2761	5.682 (2.377–13.583)	3.940 (1.639–9.472)	3.900 (1.622–9.376)	Current and <15	147,675	420	1189570.27	0.3531	1.548 (1.401–1.711)	1.647 (1.485–1.828)	1.644 (1.482–1.824)	Current and ≥15	2,396	13	18775.56	0.6924	3.048 (1.768–5.253)	2.147 (1.242–3.711)	2.101 (1.215–3.632)

Model 1, non-adjusted; Model 2, adjusted for age, sex, BMI, alcohol consumption, regular exercise, income, pneumonia, asthma; Model 3, adjusted for age, sex, BMI, alcohol consumption, regular exercise, income, pneumonia, asthma, diabetes mellitus, hypertension, dyslipidemia. *Sex was excluded from multivariate models. COPD, chronic obstructive pulmonary disease; IR, incidence rate; PY, person-year; HR, hazard ratio; CI, confidence interval; Ref., reference.

## Discussion

We found that cigarette smoking was associated with an increased risk for COPD development in young individuals. Ever-smokers had a higher risk of developing COPD than non-smokers, and the risk was the highest in current smokers. A positive dose relationship was also noted between cumulative smoking amount and COPD development. Female smokers had a higher risk of developing COPD than male smokers. Taken together, cigarette smoking is an important risk factor for COPD development in young individuals.

As for every other disease, clinical suspicion is important for COPD, which is generally diagnosed in elderly people (2). However, some individuals aged <50 years could have airflow limitations and were diagnosed with COPD (4–6). The prevalence and incidence of spirometry- and physician-diagnosed COPD in young individuals have been reported in some cohort studies. In the European Community Respiratory Health Survey, the prevalence and incidence of spirometry-diagnosed COPD in a population aged 20–44 years were 3.6% and 2.88/1000 PY, respectively (7, 12). In north-eastern Italy, the prevalence of spirometry-diagnosed COPD was 4.5% in a population aged 26–44 years (13). In Netherlands, the incidence of physician-diagnosed COPD was 0.78/1000 PY in a population aged 40–44 years (11). In this study, the incidence of physician-diagnosed COPD was 0.2617/1000 PY in individuals aged 20–39 years, which was lower than those noted in previous studies (7, 11). In South Korea, the prevalence of spirometry-diagnosed COPD in a population aged 40–50 years was 4.2%; however, only 6.3% of them were provided an inhaler for treatment (24). This implied that >90% of potential patients with COPD were not properly diagnosed by physicians in South Korea. Therefore, many young patients with COPD in South Korea were not properly managed by physicians, owing to underdiagnosis. Further active case findings with spirometry and medical management are required in this age group.

Advanced age and cigarette smoking are important risk factors for developing COPD (2, 25, 26). Particularly, cigarette smoking has been proposed as a main cause of COPD in young populations (7, 8). In this study, ever-smokers had a higher risk of developing COPD than non-smokers. Furthermore, the risk of developing COPD increased with an increase in amount of cigarette smoking. This correlation indicated cigarette smoking to be an important risk factor for COPD development in young populations.

In this study, the >10 pack-years of smoking history among individuals suggested that many of them possibly started cigarette smoking as an adolescent or during early adulthood. Although the smoking prevalence in adolescents has declined recently, many adolescents have started using e-cigarettes at an early age (27). The use of e-cigarettes was associated with an increased incidence of combustible cigarette smoking, regarded as a gateway to combustible cigarette smoking (28, 29). In the US, 22% of teenagers smoked e-cigarettes in 2020 (30), and the number of smokers in early adulthood (ages 18–23 years) has doubled between 2002 and 2018 (10). Furthermore, adolescent smokers could have significant airflow limitations, and lung function impairments might be long-term and irreversible later in life (31, 32). Younger patients with COPD had a severity distribution and progression similar to those of older patients (4). Younger patients with COPD frequently exhibit chronic respiratory symptoms, impaired lung functions, and an increased risk of hospitalization and death (6). Moreover, younger patients reported a higher impact of COPD on wellbeing, daily activities, and requirement for adjusting their activities because of their symptoms, compared to older patients (33). Therefore, multi-dimensional efforts should be considered for preventing adolescents and young adults from smoking cigarettes and e-cigarettes.

In this study, female smokers exhibited a higher risk of developing COPD than male smokers. This suggested that more vigilant monitoring of COPD is required for female smokers. COPD is considered to primarily be associated with men (1, 2); however, the proportion of former and current smokers in women has reached 28 and 17%, respectively (34). Compared to male smokers, female smokers are more likely to experience a decline in lung function (35). The rates of lung function decline have been recorded to be 0.98%/pack-year in men and 1.21%/pack-year in women (36). Female smokers have been reported to exhibit a higher risk of both developing COPD and being hospitalized due to COPD (35, 37) and a higher prevalence of severe COPD, compared to male smokers (38). The underlying mechanisms remain unclear but may be associated with the smaller lung size in women, compared to men (35). Furthermore, female sex hormones and reproductive history may be associated with lung function and COPD (39).

There were some limitations of our study. First, owing to the limitations of the original data, the diagnosis of COPD was based on the health insurance claims, and lung function could not be evaluated. To overcome this limitation, we strictly defined COPD diagnosis based on the insurance claims of 3 times/year over 2 years. With this strict definition, patients with regular hospital visits for COPD were selected, and a significant relationship was demonstrated between smoking and COPD development. Second, every potential risk factor for COPD development could not be obtained from the database. However, cigarette smoking was also significantly associated with COPD development after adjusting for important risk factors, such as age, sex, BMI, and history of respiratory diseases (1, 2, 7, 8).

## Conclusion

In conclusion, cigarette smoking is an important risk factor for developing COPD in young individuals. Current and heavy smokers exhibited higher risks of developing COPD. Female smokers were more likely to develop COPD than male smokers. Our findings suggest that active case findings and smoking cessation policies are also important for proper management and prevention of COPD in young individuals.

## Data availability statement

Publicly available datasets were analyzed in this study. This data can be found here: the Korean National Health Information Database.

## Ethics statement

The studies involving human participants were reviewed and approved by the Institutional Review Board of the Asan Medical Center, Seoul, Republic of Korea. Written informed consent for participation was not required for this study in accordance with the national legislation and the institutional requirements.

## Author contributions

CC, KL, KH, DS, and SL conceived and designed the study and contributed to data interpretation. KL and KH contributed to the data collection and data analysis. CC, DS, and SL drafted the manuscript. All authors contributed to the article and approved the submitted version.

## Funding

This study was supported by a grant from the Korea Health Promotion R&D Project, funded by the Ministry of Health and Welfare, Republic of Korea (grant number: HS21C0096), and the Bio & Medical Technology Development Program of the National Research Foundation (NRF) & funded by the Korean government (MSIT) (No. 2022M3A9G8017220).

## Conflict of interest

The authors declare that the research was conducted in the absence of any commercial or financial relationships that could be construed as a potential conflict of interest.

## Publisher’s note

All claims expressed in this article are solely those of the authors and do not necessarily represent those of their affiliated organizations, or those of the publisher, the editors and the reviewers. Any product that may be evaluated in this article, or claim that may be made by its manufacturer, is not guaranteed or endorsed by the publisher.

## Supplementary material

The Supplementary material for this article can be found online at: https://www.frontiersin.org/articles/10.3389/fmed.2023.1190885/full#supplementary-material

## Abbreviations

BMI: body-mass index
CI: confidence interval
COPD: chronic obstructive pulmonary disease
e-cigarette: electronic cigarette
HR: hazard ratio
ICD-10: International Classification of Diseases 10th Revision
NHID: National Health Information Database
NHIS: National Health Insurance Service
PY: person-year
SD: standard deviation
