# Supplementary material for: Effect of smoking on the development of chronic obstructive pulmonary disease in young individuals: a nationwide cohort study
Source: Front Med (Lausanne). 2023 Aug 1;10:1190885. doi: 10.3389/fmed.2023.1190885 (PMC10428618; doi:10.3389/fmed.2023.1190885)
Supplement: Supplementary file 1 [file Table_1.docx]

Supplementary Material

**Effect of smoking on the development of chronic obstructive pulmonary disease in young individuals: a nationwide cohort study**

Chiwook Chung^1, 2^, Kyu Na Lee^3^, Kyungdo Han^3^, Dong Wook Shin^4^*†, and Sei Won Lee^1^*†

^1^Department of Pulmonary and Critical Care Medicine, Asan Medical Center, University of Ulsan College of Medicine, Seoul, Republic of Korea.

^2^Department of Pulmonary, Allergy, and Critical Care Medicine, Gangneung Asan Hospital, University of Ulsan College of Medicine, Gangneung, Republic of Korea.

^3^Department of Statistics and Actuarial Science, Soongsil University, Seoul, Republic of Korea.

^4^Supportive Care Center, Samsung Comprehensive Cancer Center/Department of Family Medicine, Samsung Medical Center, Sungkyunkwan University School of Medicine, Seoul, Republic of Korea.

*†These authors contributed equally to this work and share last authorship

**Corresponding authors:**

**Dong Wook Shin, MD, DrPH, MBA**

Department of Family Medicine and Supportive Care Center, Samsung Medical Center, Sungkyunkwan University School of Medicine, Department of Clinical Research Design and Evaluation, Samsung Advanced Institute for Health Science and Technology (SAIHST), Sungkyunkwan University 81 Irwon-Ro, Gangnam-gu, Seoul 06351, Republic of Korea.

Tel: (82) 2-3410-5252; Fax: (82) 2-3410-0388; E-mail: dwshin.md@gmail.com

**Sei Won Lee, M.D., Ph.D.**

Department of Pulmonary and Critical Care Medicine, Asan Medical Center, University of Ulsan College of Medicine, 88 Olympic-ro 43-gil, Songpa-gu, Seoul 05505, Republic of Korea

Tel.: (82) 2-3010-3990, Fax: (82) 2-3010-6968, E-mail: [iseiwon@gmail.com](mailto:iseiwon@gmail.com)

**Supplementary Table 1.** Baseline characteristics of study population.

|  | Total |  |  |  | Male |  |  | Female |  |  |
| --- | --- | --- | --- | --- | --- | --- | --- | --- | --- | --- |
|  | Total | Non-COPD | COPD | p-value | Non-COPD | COPD | p-value | Non-COPD | COPD | p-value |
| N | 6307576 | 6293787 | 13789 |  | 3726178 | 8705 |  | 2567609 | 5084 |  |
| Age, years | 30.84±5 | 30.84±5 | 32.6±4.72 | <.0001 | 31.57±4.69 | 33.36±4.32 | <.0001 | 29.77±5.23 | 31.31±5.08 | <.0001 |
| Age groups |  |  |  | <.0001 |  |  | <.0001 |  |  | <.0001 |
| <30 years | 2669087 (42.32) | 2665337 (42.35) | 3750 (27.2) |  | 1341230 (35.99) | 1843 (21.17) |  | 1324107 (51.57) | 1907 (37.51) |  |
| ≥30 years | 3638489 (57.68) | 3628450 (57.65) | 10039 (72.8) |  | 2384948 (64.01) | 6862 (78.83) |  | 1243502 (48.43) | 3177 (62.49) |  |
| Sex |  |  |  | <.0001 |  | | |  | | |
| Male | 3734883 (59.21) | 3726178 (59.2) | 8705 (63.13) |  |  |  |  |  |  |  |
| Female | 2572693 (40.79) | 2567609 (40.8) | 5084 (36.87) |  |  |  |  |  |  |  |
| BMI, kg/m^2^ | 22.99±3.61 | 22.99±3.61 | 23.17±3.88 | <.0001 | 24.12±3.41 | 23.91±3.76 | <.0001 | 21.36±3.25 | 21.92±3.76 | <.0001 |
| Income, lowest Q1* | 1367132 (21.67) | 1364211 (21.68) | 2921 (21.18) | 0.1613 | 613776 (16.47) | 1321 (15.18) | 0.0011 | 750435 (29.23) | 1600 (31.47) | 0.0004 |
| Smoking status |  |  |  | <.0001 |  |  | <.0001 |  |  | <.0001 |
| Non | 3485089 (55.25) | 3478462 (55.27) | 6627 (48.06) |  | 1146342 (30.76) | 2192 (25.18) |  | 2332120 (90.83) | 4435 (87.23) |  |
| Former | 633122 (10.04) | 631586 (10.04) | 1536 (11.14) |  | 545735 (14.65) | 1320 (15.16) |  | 85851 (3.34) | 216 (4.25) |  |
| Current | 2189365 (34.71) | 2183739 (34.7) | 5626 (40.8) |  | 2034101 (54.59) | 5193 (59.66) |  | 149638 (5.83) | 433 (8.52) |  |
| Alcohol consumption |  |  |  | <.0001 |  |  | <.0001 |  |  | <.0001 |
| Non | 2388341 (37.86) | 2382782 (37.86) | 5559 (40.31) |  | 980980 (26.33) | 2604 (29.91) |  | 1401802 (54.6) | 2955 (58.12) |  |
| Mild | 3364896 (53.35) | 3357914 (53.35) | 6982 (50.63) |  | 2253076 (60.47) | 4978 (57.19) |  | 1104838 (43.03) | 2004 (39.42) |  |
| Heavy | 554339 (8.79) | 553091 (8.79) | 1248 (9.05) |  | 492122 (13.21) | 1123 (12.9) |  | 60969 (2.37) | 125 (2.46) |  |
| Regular exercise | 807860 (12.81) | 806090 (12.81) | 1770 (12.84) | 0.92 | 560639 (15.05) | 1273 (14.62) | 0.2711 | 245451 (9.56) | 497 (9.78) | 0.6004 |
| Diabetes mellitus | 122207 (1.94) | 121789 (1.94) | 418 (3.03) | <.0001 | 97383 (2.61) | 328 (3.77) | <.0001 | 24406 (0.95) | 90 (1.77) | <.0001 |
| Hypertension | 2239408 (35.5) | 2234091 (35.5) | 5317 (38.56) | <.0001 | 1737632 (46.63) | 4189 (48.12) | 0.0054 | 496459 (19.34) | 1128 (22.19) | <.0001 |
| Dyslipidemia | 433783 (6.88) | 432558 (6.87) | 1225 (8.88) | <.0001 | 336618 (9.03) | 966 (11.1) | <.0001 | 95940 (3.74) | 259 (5.09) | <.0001 |
| CKD | 170405 (2.7) | 170032 (2.7) | 373 (2.71) | 0.98 | 99032 (2.66) | 237 (2.72) | 0.7072 | 71000 (2.77) | 136 (2.68) | 0.6953 |
| Asthma | 182672 (2.9) | 180518 (2.87) | 2154 (15.62) | <.0001 | 74602 (2) | 1188 (13.65) | <.0001 | 105916 (4.13) | 966 (19) | <.0001 |
| Pneumonia | 26548 (0.42) | 26293 (0.42) | 255 (1.85) | <.0001 | 13708 (0.37) | 157 (1.8) | <.0001 | 12585 (0.49) | 98 (1.93) | <.0001 |

Data are presented as the mean ± standard deviation or number (%), unless otherwise indicated.

*First quartile of medical insurance premiums and medical aid beneficiaries.

COPD, chronic obstructive pulmonary disease; BMI, body mass index; CKD, chronic kidney disease.

CKD was defined by either a record of ICD-10 codes N18–19 or an estimated glomerular filtration rate of <60 mL/min/1.73 m^2^ using the Modification of Diet in Renal Disease equation in the health check-up data.

**Supplementary Table 2.** Impact of smoking status on COPD development - detailed hazard risks of covariates in Model 2 and 3.

|  | Total, HR (95% CI) | | Male*, HR (95% CI) | | Female*, HR (95% CI) | |
| --- | --- | --- | --- | --- | --- | --- |
|  | Model 2 | Model 3 | Model 2 | Model 3 | Model 2 | Model 3 |
| Smoking status |  |  |  |  |  |  |
| Non | 1 (Ref.) | 1 (Ref.) | 1 (Ref.) | 1 (Ref.) | 1 (Ref.) | 1 (Ref.) |
| Former | 1.208 (1.135–1.285) | 1.206 (1.134–1.283) | 1.116 (1.041–1.197) | 1.115 (1.040–1.195) | 1.389 (1.210–1.595) | 1.388 (1.210–1.594) |
| Current | 1.460 (1.394–1.529) | 1.455 (1.389–1.524) | 1.395 (1.324–1.469) | 1.390 (1.320–1.464) | 1.658 (1.497–1.837) | 1.654 (1.493–1.833) |
| Age, years | 1.063 (1.059–1.067) | 1.062 (1.058–1.066) | 1.080 (1.074–1.085) | 1.078 (1.073–1.083) | 1.045 (1.039–1.050) | 1.044 (1.038–1.050) |
| Sex |  |  |  |  |  |  |
| Male | 1.018 (0.971–1.067) | 1.009 (0.962–1.058) |  | |  | |
| Female | 1 (Ref.) | 1 (Ref.) |  |  |  |  |
| BMI, kg/m^2^ | 0.990 (0.985–0.995) | 0.986 (0.981–0.992) | 0.968 (0.962–0.974) | 0.964 (0.958–0.970) | 1.028 (1.020–1.036) | 1.025 (1.016–1.033) |
| Alcohol consumption |  |  |  |  |  |  |
| Non | 1 (Ref.) | 1 (Ref.) | 1 (Ref.) | 1 (Ref.) | 1 (Ref.) | 1 (Ref.) |
| Mild | 0.857 (0.825–0.890) | 0.856 (0.825–0.889) | 0.815 (0.777–0.856) | 0.814 (0.776–0.855) | 0.907 (0.855–0.961) | 0.908 (0.856–0.962) |
| Heavy | 0.851 (0.797–0.908) | 0.845 (0.791–0.902) | 0.827 (0.770–0.889) | 0.821 (0.764–0.883) | 0.925 (0.768–1.113) | 0.923 (0.767–1.110) |
| Regular exercise | 0.978 (0.930–1.028) | 0.979 (0.931–1.030) | 0.981 (0.924–1.041) | 0.983 (0.926–1.043) | 0.972 (0.886–1.067) | 0.973 (0.887–1.068) |
| Income |  |  |  |  |  |  |
| Q2-4 | 1 (Ref.) | 1 (Ref.) | 1 (Ref.) | 1 (Ref.) | 1 (Ref.) | 1 (Ref.) |
| Q1 (lowest) | 1.092 (1.047–1.138) | 1.090 (1.045–1.137) | 1.117 (1.052–1.186) | 1.114 (1.049–1.182) | 1.064 (1.002–1.129) | 1.062 (1.000–1.127) |
| Pneumonia | 3.335 (2.945–3.778) | 3.325 (2.936–3.766) | 3.618 (3.087–4.239) | 3.606 (3.077–4.226) | 2.921 (2.389–3.570) | 2.910 (2.381–3.558) |
| Asthma | 5.943 (5.669–6.229) | 5.944 (5.671–6.230) | 6.975 (6.556–7.421) | 6.977 (6.557–7.423) | 4.899 (4.563–5.260) | 4.896 (4.559–5.256) |
| Diabetes Mellitus |  | 1.262 (1.143–1.394) |  | 1.226 (1.096–1.371) |  | 1.341 (1.084–1.659) |
| Hypertension |  | 1.043 (1.005–1.082) |  | 1.047 (1.002–1.093) |  | 1.052 (0.982–1.126) |
| Dyslipidemia |  | 1.105 (1.040–1.174) |  | 1.103 (1.029–1.181) |  | 1.130 (0.994–1.284) |

Model 2: adjusted for age, sex, BMI, alcohol consumption, regular exercise, income, pneumonia, asthma.

Model 3: adjusted for age, sex, BMI, alcohol consumption, regular exercise, income, pneumonia, asthma, diabetes mellitus, hypertension, dyslipidemia.

*Sex was excluded from multivariate models.

COPD, chronic obstructive pulmonary disease; HR, hazard ratio; CI, confidence interval; Ref., reference; BMI, body mass index.

**Supplementary Table 3.** Impact of smoking status on COPD development - Subgroup analysis for interaction.

|  | Smoking status | Total | | | | | | Male* | | | | |  | Female* | | | | |  |
| --- | --- | --- | --- | --- | --- | --- | --- | --- | --- | --- | --- | --- | --- | --- | --- | --- | --- | --- | --- |
|  |  | n | Event | Duration | IR | HR (95% CI) | p-value for interaction | n | Event | Duration | IR | HR (95% CI) | p-value for interaction | n | Event | Duration | IR | HR (95% CI) | p-value for interaction |
| Age groups |  |  |  |  |  |  | <.0001 |  |  |  |  |  | 0.0465 |  |  |  |  |  | 0.0291 |
| <30 years | Non | 1685294 | 2236 | 13867621.18 | 0.1612 | 1 (Ref.) |  | 492654 | 577 | 4011994.3 | 0.1438 | 1 (Ref.) |  | 1192640 | 1659 | 9855626.87 | 0.1683 | 1 (Ref.) |  |
|  | Former | 175135 | 266 | 1447186.8 | 0.1838 | 1.183 (1.040–1.346) |  | 129350 | 193 | 1073464.41 | 0.1798 | 1.256 (1.066–1.478) |  | 45785 | 73 | 373722.39 | 0.1953 | 1.153 (0.911–1.458) |  |
|  | Current | 808658 | 1248 | 6659720.57 | 0.1874 | 1.260 (1.168–1.358) |  | 721069 | 1073 | 5949999.62 | 0.1803 | 1.320 (1.192–1.461) |  | 87589 | 175 | 709720.95 | 0.2466 | 1.465 (1.250–1.717) |  |
| ≥30 years | Non | 1799795 | 4391 | 15095773.36 | 0.2909 | 1 (Ref.) |  | 655880 | 1615 | 5593136.48 | 0.2888 | 1 (Ref.) |  | 1143915 | 2776 | 9502636.88 | 0.2921 | 1 (Ref.) |  |
|  | Former | 457987 | 1270 | 3919980.5 | 0.3240 | 1.229 (1.148–1.316) |  | 417705 | 1127 | 3596957.48 | 0.3133 | 1.098 (1.017–1.187) |  | 40282 | 143 | 323023.01 | 0.4427 | 1.531 (1.293–1.813) |  |
|  | Current | 1380707 | 4378 | 11709371.51 | 0.3739 | 1.538 (1.461–1.620) |  | 1318225 | 4120 | 11210746.63 | 0.3675 | 1.415 (1.334–1.502) |  | 62482 | 258 | 498624.88 | 0.5174 | 1.791 (1.573–2.041) |  |
| BMI |  |  |  |  |  |  | <.0001 |  |  |  |  |  | 0.0011 |  |  |  |  |  | 0.4589 |
| <25 kg/m^2^ | Non | 2833251 | 5123 | 23553656.42 | 0.2175 | 1 (Ref.) |  | 764025 | 1402 | 6388188.32 | 0.2195 | 1 (Ref.) |  | 2069226 | 3721 | 17165468.11 | 0.2168 | 1 (Ref.) |  |
|  | Former | 406513 | 1014 | 3444765.21 | 0.2944 | 1.337 (1.243–1.439) |  | 333225 | 850 | 2850726.64 | 0.2982 | 1.214 (1.113–1.323) |  | 73288 | 164 | 594038.57 | 0.2761 | 1.328 (1.134–1.554) |  |
|  | Current | 1401202 | 3714 | 11757971.21 | 0.3159 | 1.608 (1.524–1.695) |  | 1276869 | 3380 | 10755555.47 | 0.3143 | 1.476 (1.385–1.574) |  | 124333 | 334 | 1002415.74 | 0.3332 | 1.662 (1.481–1.865) |  |
| ≥25 kg/m^2^ | Non | 651838 | 1504 | 5409738.11 | 0.2780 | 1 (Ref.) |  | 384509 | 790 | 3216942.47 | 0.2456 | 1 (Ref.) |  | 267329 | 714 | 2192795.64 | 0.3256 | 1 (Ref.) |  |
|  | Former | 226609 | 522 | 1922402.08 | 0.2715 | 0.968 (0.874–1.072) |  | 213830 | 470 | 1819695.25 | 0.2583 | 0.958 (0.854–1.075) |  | 12779 | 52 | 102706.83 | 0.5063 | 1.623 (1.224–2.152) |  |
|  | Current | 788163 | 1912 | 6611120.87 | 0.2892 | 1.182 (1.101–1.269) |  | 762425 | 1813 | 6405190.78 | 0.2831 | 1.247 (1.146–1.357) |  | 25738 | 99 | 205930.09 | 0.4808 | 1.633 (1.320–2.019) |  |
| Alcohol consumption |  |  |  |  |  |  | 0.0008 |  |  |  |  |  | 0.2685 |  |  |  |  |  | 0.7039 |
| Non | Non | 1827459 | 3811 | 15207548.29 | 0.2506 | 1 (Ref.) |  | 487892 | 1059 | 4082739.35 | 0.2594 | 1 (Ref.) |  | 1339567 | 2752 | 11124808.95 | 0.2474 | 1 (Ref.) |  |
|  | Former | 141869 | 437 | 1196883.82 | 0.3651 | 1.353 (1.221–1.499) |  | 115102 | 356 | 979858.48 | 0.3633 | 1.196 (1.061–1.349) |  | 26767 | 81 | 217025.34 | 0.3732 | 1.488 (1.193–1.856) |  |
|  | Current | 419013 | 1311 | 3503982.05 | 0.3742 | 1.575 (1.470–1.688) |  | 380590 | 1189 | 3194290.38 | 0.3722 | 1.450 (1.335–1.575) |  | 38423 | 122 | 309691.68 | 0.3939 | 1.615 (1.347–1.937) |  |
| Mild, Heavy | Non | 1657630 | 2816 | 13755846.24 | 0.2047 | 1 (Ref.) |  | 660642 | 1133 | 5522391.44 | 0.2052 | 1 (Ref.) |  | 996988 | 1683 | 8233454.8 | 0.2044 | 1 (Ref.) |  |
|  | Former | 491253 | 1099 | 4170283.47 | 0.2635 | 1.131 (1.050–1.217) |  | 431953 | 964 | 3690563.41 | 0.2612 | 1.071 (0.982–1.167) |  | 59300 | 135 | 479720.07 | 0.2814 | 1.335 (1.120–1.592) |  |
|  | Current | 1770352 | 4315 | 14865110.02 | 0.2903 | 1.391 (1.318–1.469) |  | 1658704 | 4004 | 13966455.87 | 0.2867 | 1.353 (1.266–1.447) |  | 111648 | 311 | 898654.15 | 0.3461 | 1.675 (1.479–1.896) |  |
| Regular exercise | |  |  |  |  |  | 0.1796 |  |  |  |  |  | 0.4327 |  |  |  |  |  | 0.422 |
| No | Non | 3090257 | 5854 | 25674515.53 | 0.2280 | 1 (Ref.) |  | 975035 | 1856 | 8151940.82 | 0.2277 | 1 (Ref.) |  | 2115222 | 3998 | 17522574.71 | 0.2282 | 1 (Ref.) |  |
|  | Former | 519385 | 1276 | 4393606.87 | 0.2904 | 1.233 (1.153–1.318) |  | 442949 | 1081 | 3774878.92 | 0.2864 | 1.138 (1.054–1.228) |  | 76436 | 195 | 618727.96 | 0.3152 | 1.420 (1.229–1.642) |  |
|  | Current | 1890074 | 4889 | 15850035.13 | 0.3085 | 1.474 (1.404–1.548) |  | 1754987 | 4495 | 14762432.87 | 0.3045 | 1.409 (1.332–1.490) |  | 135087 | 394 | 1087602.26 | 0.3623 | 1.687 (1.516–1.878) |  |
| Yes | Non | 394832 | 773 | 3288879 | 0.2350 | 1 (Ref.) |  | 173499 | 336 | 1453189.96 | 0.2312 | 1 (Ref.) |  | 221333 | 437 | 1835689.04 | 0.2381 | 1 (Ref.) |  |
|  | Former | 113737 | 260 | 973560.42 | 0.2671 | 1.084 (0.940–1.250) |  | 104106 | 239 | 895542.98 | 0.2669 | 1.013 (0.858–1.197) |  | 9631 | 21 | 78017.44 | 0.2692 | 1.151 (0.743–1.784) |  |
|  | Current | 299291 | 737 | 2519056.95 | 0.2926 | 1.368 (1.232–1.518) |  | 284307 | 698 | 2398313.38 | 0.2910 | 1.316 (1.155–1.500) |  | 14984 | 39 | 120743.57 | 0.3230 | 1.412 (1.016–1.961) |  |
| Asthma |  |  |  |  |  |  | <.0001 |  |  |  |  |  | 0.9308 |  |  |  |  |  | 0.1407 |
| No | Non | 3362197 | 5457 | 27962057.61 | 0.1952 | 1 (Ref.) |  | 1122018 | 1838 | 9385088.89 | 0.1958 | 1 (Ref.) |  | 2240179 | 3619 | 18576968.72 | 0.1948 | 1 (Ref.) |  |
|  | Former | 614245 | 1256 | 5210506.8 | 0.2411 | 1.156 (1.081–1.236) |  | 532171 | 1090 | 4545095.67 | 0.2398 | 1.123 (1.040–1.212) |  | 82074 | 166 | 665411.13 | 0.2495 | 1.342 (1.148–1.570) |  |
|  | Current | 2148462 | 4922 | 18033781.09 | 0.2729 | 1.402 (1.336–1.472) |  | 2004904 | 4589 | 16876202.66 | 0.2719 | 1.398 (1.322–1.478) |  | 143558 | 333 | 1157578.43 | 0.2877 | 1.584 (1.411–1.778) |  |
| Yes | Non | 122892 | 1170 | 1001336.93 | 1.1684 | 1 (Ref.) |  | 26516 | 354 | 220041.9 | 1.6088 | 1 (Ref.) |  | 96376 | 816 | 781295.03 | 1.0444 | 1 (Ref.) |  |
|  | Former | 18877 | 280 | 156660.49 | 1.7873 | 1.486 (1.301–1.699) |  | 14884 | 230 | 125326.22 | 1.8352 | 1.084 (0.918–1.280) |  | 3993 | 50 | 31334.27 | 1.5957 | 1.575 (1.183–2.097) |  |
|  | Current | 40903 | 704 | 335310.99 | 2.0995 | 1.868 (1.693–2.061) |  | 34390 | 604 | 284543.59 | 2.1227 | 1.379 (1.208–1.573) |  | 6513 | 100 | 50767.4 | 1.9698 | 1.967 (1.595–2.425) |  |
| Pneumonia |  |  |  |  |  |  | 0.2149 |  |  |  |  |  | 0.0128 |  |  |  |  |  | 0.5882 |
| No | Non | 3469584 | 6486 | 28837389.74 | 0.2249 | 1 (Ref.) |  | 1144407 | 2137 | 9571536 | 0.2233 | 1 (Ref.) |  | 2325177 | 4349 | 19265853.74 | 0.2257 | 1 (Ref.) |  |
|  | Former | 630048 | 1498 | 5341568.07 | 0.2804 | 1.203 (1.131–1.281) |  | 544474 | 1287 | 4648713.32 | 0.2769 | 1.120 (1.043–1.202) |  | 85574 | 211 | 692854.75 | 0.3045 | 1.390 (1.209–1.598) |  |
|  | Current | 2181396 | 5550 | 18303241.02 | 0.3032 | 1.464 (1.397–1.534) |  | 2032137 | 5124 | 17101215.44 | 0.2996 | 1.408 (1.336–1.484) |  | 149259 | 426 | 1202025.58 | 0.3544 | 1.671 (1.507–1.853) |  |
| Yes | Non | 15505 | 141 | 126004.79 | 1.1190 | 1 (Ref.) |  | 4127 | 55 | 33594.78 | 1.6372 | 1 (Ref.) |  | 11378 | 86 | 92410.01 | 0.9306 | 1 (Ref.) |  |
|  | Former | 3074 | 38 | 25599.22 | 1.4844 | 1.415 (0.988–2.027) |  | 2581 | 33 | 21708.57 | 1.5201 | 0.941 (0.611–1.449) |  | 493 | 5 | 3890.65 | 1.2851 | 1.347 (0.547–3.319) |  |
|  | Current | 7969 | 76 | 65851.06 | 1.1541 | 1.228 (0.928–1.626) |  | 7157 | 69 | 59530.81 | 1.1591 | 0.831 (0.583–1.185) |  | 812 | 7 | 6320.25 | 1.1076 | 1.111 (0.514–2.402) |  |
| Asthma or  Pneumonia | | |  |  |  |  | <.0001 |  |  |  |  |  | 0.739 |  |  |  |  |  | 0.1675 |
| No | Non | 3348642 | 5390 | 27851462.85 | 0.1935 | 1 (Ref.) |  | 1118283 | 1810 | 9354616.7 | 0.1935 | 1 (Ref.) |  | 2230359 | 3580 | 18496846.16 | 0.1936 | 1 (Ref.) |  |
|  | Former | 611480 | 1237 | 5187338.06 | 0.2385 | 1.154 (1.079–1.235) |  | 529818 | 1074 | 4525211.68 | 0.2373 | 1.128 (1.044–1.218) |  | 81662 | 163 | 662126.38 | 0.2462 | 1.335 (1.140–1.563) |  |
|  | Current | 2141142 | 4877 | 17973133.7 | 0.2714 | 1.403 (1.337–1.473) |  | 1998253 | 4547 | 16820811.6 | 0.2703 | 1.406 (1.329–1.487) |  | 142889 | 330 | 1152322.09 | 0.2864 | 1.588 (1.414–1.784) |  |
| Yes | Non | 136447 | 1237 | 1111931.68 | 1.1125 | 1 (Ref.) |  | 30251 | 382 | 250514.09 | 1.5249 | 1 (Ref.) |  | 106196 | 855 | 861417.59 | 0.9926 | 1 (Ref.) |  |
|  | Former | 21642 | 299 | 179829.23 | 1.6627 | 1.476 (1.297–1.681) |  | 17237 | 246 | 145210.21 | 1.6941 | 1.066 (0.908–1.252) |  | 4405 | 53 | 34619.02 | 1.5310 | 1.591 (1.205–2.101) |  |
|  | Current | 48223 | 749 | 395958.38 | 1.8916 | 1.820 (1.654–2.004) |  | 41041 | 646 | 339934.65 | 1.9004 | 1.337 (1.177–1.518) |  | 7182 | 103 | 56023.73 | 1.8385 | 1.928 (1.569–2.371) |  |

Model 2: adjusted for age, sex, BMI, alcohol consumption, regular exercise, income, pneumonia, asthma.

*Sex was excluded from multivariate models.

COPD, chronic obstructive pulmonary disease; IR, incidence rate (per 1000 person-year); Ref., reference; BMI, body mass index.

**Supplementary Table 4.** Impact of smoking amount on COPD development - detailed hazard risks of covariates in Model 2 and 3.

|  | Total, HR (95% CI) | | Male*, HR (95% CI) | | Female*, HR (95% CI) | |
| --- | --- | --- | --- | --- | --- | --- |
|  | Model 2 | Model 3 | Model 2 | Model 3 | Model 2 | Model 3 |
| Smoking amount,  Pack-year |  |  |  |  |  |  |
| Non | 1 (Ref.) | 1 (Ref.) | 1 (Ref.) | 1 (Ref.) | 1 (Ref.) | 1 (Ref.) |
| <10 | 1.266 (1.206–1.328) | 1.264 (1.205–1.327) | 1.177 (1.113–1.244) | 1.175 (1.112–1.242) | 1.508 (1.380–1.648) | 1.506 (1.378–1.646) |
| 10≤ …<20 | 1.548 (1.461–1.639) | 1.539 (1.453–1.631) | 1.456 (1.370–1.547) | 1.450 (1.365–1.541) | 2.346 (1.749–3.146) | 2.320 (1.730–3.112) |
| ≥20 | 2.236 (2.062–2.425) | 2.209 (2.037–2.396) | 2.067 (1.899–2.249) | 2.049 (1.883–2.231) | 3.265 (1.627–6.552) | 3.193 (1.591–6.408) |
| Age, years | 1.055 (1.051–1.059) | 1.054 (1.050–1.058) | 1.066 (1.060–1.071) | 1.064 (1.059–1.070) | 1.044 (1.038–1.050) | 1.043 (1.038–1.049) |
| Sex |  |  |  |  |  |  |
| Male | 1.009 (0.962–1.058) | 1.001 (0.954–1.050) |  | |  | |
| Female | 1 (Ref.) | 1 (Ref.) |  |  |  |  |
| BMI, kg/m^2^ | 0.989 (0.984–0.994) | 0.986 (0.981–0.991) | 0.966 (0.960–0.972) | 0.962 (0.956–0.969) | 1.028 (1.021–1.037) | 1.025 (1.016–1.033) |
| Alcohol consumption |  |  |  |  |  |  |
| Non | 1 (Ref.) | 1 (Ref.) | 1 (Ref.) | 1 (Ref.) | 1 (Ref.) | 1 (Ref.) |
| Mild | 0.859 (0.828–0.892) | 0.859 (0.827–0.892) | 0.822 (0.782–0.863) | 0.821 (0.781–0.862) | 0.908 (0.857–0.962) | 0.909 (0.857–0.963) |
| Heavy | 0.810 (0.758–0.865) | 0.806 (0.754–0.861) | 0.788 (0.733–0.848) | 0.783 (0.728–0.843) | 0.916 (0.761–1.103) | 0.915 (0.760–1.101) |
| Regular exercise | 0.978 (0.930–1.028) | 0.979 (0.931–1.029) | 0.978 (0.922–1.038) | 0.980 (0.923–1.040) | 0.971 (0.885–1.066) | 0.973 (0.887–1.067) |
| Income |  |  |  |  |  |  |
| Q2-4 | 1 (Ref.) | 1 (Ref.) | 1 (Ref.) | 1 (Ref.) | 1 (Ref.) | 1 (Ref.) |
| Q1 (lowest) | 1.098 (1.053–1.145) | 1.096 (1.051–1.143) | 1.111 (1.047–1.180) | 1.108 (1.044–1.176) | 1.064 (1.003–1.130) | 1.062 (1.000–1.127) |
| Pneumonia | 3.314 (2.926–3.753) | 3.305 (2.918–3.743) | 3.574 (3.050–4.189) | 3.564 (3.042–4.177) | 2.911 (2.381–3.559) | 2.900 (2.372–3.546) |
| Asthma | 5.948 (5.675–6.234) | 5.949 (5.676–6.235) | 6.946 (6.529–7.390) | 6.947 (6.530–7.391) | 4.892 (4.556–5.253) | 4.889 (4.553–5.249) |
| Diabetes Mellitus |  | 1.235 (1.118–1.364) |  | 1.205 (1.077–1.347) |  | 1.335 (1.079–1.652) |
| Hypertension |  | 1.041 (1.004–1.080) |  | 1.046 (1.001–1.092) |  | 1.052 (0.983–1.127) |
| Dyslipidemia |  | 1.090 (1.026–1.159) |  | 1.093 (1.020–1.171) |  | 1.126 (0.991–1.279) |

Model 2: adjusted for age, sex, BMI, alcohol consumption, regular exercise, income, pneumonia, asthma.

Model 3: adjusted for age, sex, BMI, alcohol consumption, regular exercise, income, pneumonia, asthma, diabetes mellitus, hypertension, dyslipidemia.

*Sex was excluded from multivariate models.

COPD, chronic obstructive pulmonary disease; HR, hazard ratio; CI, confidence interval; Ref., reference; BMI, body mass index.

**Supplementary Table 5.** Impact of smoking status and amount on COPD development - detailed hazard risks of covariates in Model 2 and 3.

|  | Total | | Male* | | Female* | |
| --- | --- | --- | --- | --- | --- | --- |
|  | Model 2 | Model 3 | Model 2 | Model 3 | Model 2 | Model 3 |
| Smoking status & amount,  Pack-year | |  |  |  |  |  |
| Non | 1 (Ref.) | 1 (Ref.) | 1 (Ref.) | 1 (Ref.) | 1 (Ref.) | 1 (Ref.) |
| Former & <15 | 1.201 (1.125–1.281) | 1.200 (1.124–1.280) | 1.104 (1.026–1.189) | 1.104 (1.026–1.188) | 1.369 (1.191–1.573) | 1.368 (1.190–1.572) |
| Former & ≥15 | 1.493 (1.297–1.718) | 1.478 (1.284–1.702) | 1.369 (1.185–1.581) | 1.360 (1.177–1.570) | 3.940 (1.639–9.472) | 3.900 (1.622–9.376) |
| Current & <15 | 1.339 (1.275–1.406) | 1.336 (1.272–1.403) | 1.262 (1.194–1.333) | 1.259 (1.192–1.330) | 1.647 (1.485–1.828) | 1.644 (1.482–1.824) |
| Current & ≥15 | 2.015 (1.888–2.150) | 1.997 (1.872–2.132) | 1.876 (1.752–2.010) | 1.865 (1.741–1.998) | 2.147 (1.242–3.711) | 2.101 (1.215–3.632) |
| Age, years | 1.057 (1.053–1.061) | 1.056 (1.052–1.060) | 1.068 (1.063–1.074) | 1.067 (1.061–1.072) | 1.044 (1.039–1.050) | 1.044 (1.038–1.049) |
| Sex |  |  |  |  |  |  |
| Male | 1.009 (0.962–1.059) | 1.001 (0.954–1.051) |  | |  | |
| Female | 1 (Ref.) | 1 (Ref.) |  |  |  |  |
| BMI, kg/m^2^ | 0.990 (0.985–0.995) | 0.986 (0.981–0.991) | 0.967 (0.961–0.973) | 0.963 (0.957–0.970) | 1.028 (1.020–1.036) | 1.025 (1.016–1.033) |
| Alcohol consumption |  |  |  |  |  |  |
| Non | 1 (Ref.) | 1 (Ref.) | 1 (Ref.) | 1 (Ref.) | 1 (Ref.) | 1 (Ref.) |
| Mild | 0.858 (0.826–0.890) | 0.857 (0.825–0.890) | 0.818 (0.779–0.859) | 0.817 (0.778–0.858) | 0.907 (0.856–0.961) | 0.908 (0.857–0.962) |
| Heavy | 0.814 (0.762–0.869) | 0.809 (0.758–0.865) | 0.792 (0.736–0.851) | 0.786 (0.731–0.846) | 0.919 (0.763–1.106) | 0.917 (0.762–1.104) |
| Regular exercise | 0.983 (0.934–1.033) | 0.984 (0.936–1.035) | 0.986 (0.929–1.047) | 0.988 (0.931–1.048) | 0.972 (0.886–1.066) | 0.973 (0.887–1.068) |
| Income |  |  |  |  |  |  |
| Q2-4 | 1 (Ref.) | 1 (Ref.) | 1 (Ref.) | 1 (Ref.) | 1 (Ref.) | 1 (Ref.) |
| Q1 (lowest) | 1.095 (1.050–1.142) | 1.093 (1.048–1.140) | 1.108 (1.044–1.177) | 1.105 (1.041–1.173) | 1.063 (1.002–1.129) | 1.061 (1.000–1.126) |
| Pneumonia | 3.326 (2.937–3.767) | 3.317 (2.929–3.757) | 3.599 (3.071–4.217) | 3.588 (3.062–4.205) | 2.915 (2.384–3.563) | 2.904 (2.376–3.550) |
| Asthma | 5.966 (5.692–6.254) | 5.967 (5.693–6.254) | 6.997 (6.577–7.444) | 6.998 (6.577–7.446) | 4.897 (4.560–5.258) | 4.893 (4.557–5.254) |
| Diabetes Mellitus |  | 1.237 (1.120–1.366) |  | 1.205 (1.077–1.348) |  | 1.339 (1.082–1.657) |
| Hypertension |  | 1.042 (1.004–1.081) |  | 1.046 (1.002–1.092) |  | 1.052 (0.982–1.126) |
| Dyslipidemia |  | 1.093 (1.029–1.162) |  | 1.096 (1.023–1.174) |  | 1.128 (0.992–1.281) |

Model 2: adjusted for age, sex, BMI, alcohol consumption, regular exercise, income, pneumonia, asthma.

Model 3: adjusted for age, sex, BMI, alcohol consumption, regular exercise, income, pneumonia, asthma, diabetes mellitus, hypertension, dyslipidemia.

*Sex was excluded from multivariate models.

COPD, chronic obstructive pulmonary disease; HR, hazard ratio; CI, confidence interval; Ref., reference; BMI, body mass index.
